# Impact of age and pre-existing influenza immune responses in humans receiving split inactivated influenza vaccine on the induction of the breadth of antibodies to influenza A strains

**DOI:** 10.1371/journal.pone.0185666

**Published:** 2017-11-01

**Authors:** Ivette A. Nuñez, Michael A. Carlock, James D. Allen, Simon O. Owino, Krissy K. Moehling, Patricia Nowalk, Michael Susick, Kensington Diagle, Kristen Sweeney, Sophia Mundle, Thorsten U. Vogel, Simon Delagrave, Moti Ramgopal, Richard K. Zimmerman, Harry Kleanthous, Ted M. Ross

**Affiliations:** 1 Center for Vaccines and Immunology, University of Georgia, Athens, Georgia, United States of America; 2 Department of Family Medicine, University of Pittsburgh, Pittsburgh, Pennsylvania, United States of America; 3 Martin Health System, Clinical Research Division, Stuart, Florida, United States of America; 4 Sanofi Pasteur, Inc., Research North America, Cambridge, Massachusetts, United States of America; 5 Department of Infectious Diseases, University of Georgia, Athens, Georgia, United States of America; University of South Dakota, UNITED STATES

## Abstract

Most humans have pre-existing immunity to influenza viruses. In this study, volunteers (ages of 18–85 years) were vaccinated with split, inactivated Fluzone^™^ influenza vaccine in four consecutive influenza seasons from 2013 to 2016 seasons. The impact of repeated vaccination on breadth and durability of antibodies was assessed as a result of vaccine strain changes. Total IgG anti-hemagglutinin (HA) binding antibodies and hemagglutination-inhibition (HAI) activity increased in all age groups against both influenza A HA components in the vaccine post-vaccination (day 21). However, younger subjects maintained seroprotective titers to the vaccine strains, which resulted in higher seroconversion rates in the elderly, since the HAI titers in elderly subjects were more likely to decline prior to the next season. Young subjects had significant HAI activity against historical, as well as contemporary H1 and H3 vaccine strains from the mid-1980s to present. In contrast, elderly subjects had HAI activity to H1 strains from all years, but were more likely to have HAI activity to older strains from 1918-1950s. They also had a more restricted HAI profile against H3 viruses compared to young subjects recognizing H3N2 influenza viruses from the mid-2000s to present. Vaccine recipients were then categorized by whether subjects seroconverted from a seronegative or seropositive pre-vaccination state. Regardless of age, immunological recall or ‘back-boosting’ to antigenically related strains were associated with seroconversion to the vaccine strain. Overall, both younger and older people have the ability to mount a breadth of immune responses following influenza vaccination. This report describes how imprinting exposure differs across age groups, influences antibody cross-reactivity to past hemagglutinin antigenic variants, and shapes immune responses elicited by current split inactivated influenza vaccines. Understanding how current influenza vaccines are influenced by pre-existing immunity in people of different ages is critical for designing the next-generation of ‘universal’ or broadly-protective influenza vaccines.

## Introduction

Influenza A virus (IAV) infection is a recurrent health and economic burden as it cycles between the human population and the animal reservoir [1]. Worldwide, there are millions of hospitalizations and thousands of deaths each year as seasonal epidemics, frequent pandemics, and spillover events together affect between 5% and 30% of the global population. The population with greatest disease severity includes children under the age of <1 and the elderly over the age of >65, with 54–70% of hospitalizations and 71–85% of deaths occurring in adults over the age of sixty-five [2]. Aging is associated with diminished immunity that impacts antibody production, induction of naïve T cell populations, and B-cell activation. In addition, aging can result in the malfunction of CD4 T cells, which are critical for sustaining the responses of innate and adaptive immune cells against invading pathogens [3] [4]. Influenza vaccine induced antibody responses in the elderly are often compromised and wane before the next season can can result in poor protection [5]. Annual influenza vaccination is recommended for these high risk groups, but has been proven to be most effective in children and young adults [6] [7]. In seasons where influenza viruses undergo change (drift or shift), the current licensed influenza vaccines available can offer variable efficacy [8]. However, antibody breadth induced post-vaccination against historical or previously circulating or antigenically related strains has the potential to increase the effectiveness of the trivalent and quadrivalent influenza vaccine (TIV or QIV) leading to increased seroprotection [9]. This study investigates the polyclonal antibody-elicited response to the annual split inactivated influenza virus (IIV) vaccine, Fluzone^™^, in a cohort of individuals that were vaccinated over 4 consecutive influenza seasons. Serum samples were collected and analyzed with the goal of determining how vaccination against currently circulating influenza viruses is influenced by both pre-existing immune responses and age-dependent seroconversion influence vaccination against currently circulating influenza viruses. In this study, we report that 1) seroconversion to the vaccine strain is essential to provide immunity against past-historical strains of influenza, also known as back-boosting, 2) that individuals can differentially respond to the different influenza A virus HA components in the vaccine, and that 3) pre-existing antibodies with hemagglutination-inhibition (HAI) activity against past vaccine strains can vary between age groups. This report focuses on the HAI results against influenza A viruses and influenza B viruses (IBV) will reported in subsequent reports.

## Materials and methods

### Ethics statement and role of the funding source

The study procedures, informed consent, and data collection documents were reviewed and approved by was approved by the Western Institutional Review Board and the Institutional Review Boards of the University of Pittsburgh and the University of Georgia. Informed written consent was obtained from the parents/guardians of the children. The funding source had no role in sample collection nor decision to submit the paper for publication.

### Subjects

Eligible volunteers between the ages of 18 and 85 years old (y.o.), who had not yet received the seasonal influenza vaccine, were enrolled beginning in September of each year. Influenza virus did not circulate widely in the community during the time periods that the subjects participated and the participants were not monitored for influenza virus infection during that time-period. These volunteers were recruited from two sites that included medical facilities in Pittsburgh, Pennsylvania and Stuart, Florida and enrolled with written, informed consent. Exclusion criteria included documented contraindications to Guillain-Barré syndrome, dementia or Alzheimer disease, allergies to eggs or egg products, estimated life expectancy <2 years, medical treatment causing or diagnosis of an immunocompromising condition, or concurrent participation in another influenza vaccine research study.

In the 2013 season (year 1), 127 eligible subjects were enrolled, in the 2014 season (year 2), 275 eligible subjects were enrolled in the 2015 season (year 3), 270 eligible subjects were enrolled, and in the 2016 final season (year 4), 270 eligible volunteers were enrolled. All northern hemisphere seasons are referred to just the autumn time-period, since vaccinations and collections occurred each year between September 1^st^ to December 15^th^ each year. Overall, in years 2–4, 225 subjects returned in Year 3 and 175 participated in all three seasons (Table 1). Each year, volunteers between the ages of 18–64 years received the standard-dose (15 μg) inactivated influenza Fluzone^™^ (Sanofi Pasteur, Swiftwater, PA, USA) vaccine (IIV-SD) and subjects over 65 years of age were offered either IIV-SD or a high dose (60 μg) Fluzone^™^ IIV (IIV-HD) before the start of the study (Table 2). The trial was approved by three institutional review boards (Western Institutional Review Board, University of Georgia, and University of Pittsburgh Institutional Review Board), and all subjects gave written informed consent. The IIV formulations consisted of three or four strains of influenza virus as specified by the U.S. Food and Drug Administration for inclusion in the vaccine each year (Table 2). For years 1 and 2, the strains included a trivalent formulation of A/California/7/2009 (H1N1), A/Texas/50/2012 (H3N2), B/Massachusetts/2/2012 (Yamagata lineage). In year 3, a quadrivalent influenza vaccine (WIV) formulation composed of A/California/7/2009 (H1N1), A/Switzerland/9715293/2013 (H3N2), B/Phuket/3073/2013 (Yamagata lineage), and B/Brisbane/60/2008 (Victoria lineage) was used for vaccination. The year 4, QIV contained the same strains as year 3 except that the H3N2 component was switched to A/Hong Kong/1/2014 (H3N2). Blood (70–90 ml) was collected from each subject at the time of vaccination (D0) and collected again 7–9 days (D7) and 21–28 days (D21) post-vaccination. Blood samples were processed for sera and peripheral blood mononuclear cells (PBMC) at all three time points and samples were aliquoted within 24 hours of collection and stored at -150°C for future analysis. In this study, sera from samples collected at days 0 and 21 were tested for the ability to mediate hemagglutination inhibition (HAI) against a panel of H1N1 and H3N2 viruses representing historical and current vaccine strains.

**Table 1:** Demographics of volunteers.

**Season**		**2013**					**2014**				
		**Total**	18–34*	**35–49**	**50–64**	**65–85**	**Total**	18–34*	**35–49**	**50–64**	**65–85**
**Subject (#)**		**127**	**28**	**4**	**46**	**49**	**275**	**45**	**38**	**100**	**92**
**Gender (%)**	**Male**	**27%**	16%	29%	29%	30%	**27%**	16%	29%	29%	30%
	**Female**	**73%**	80%	74%	70%	72%	**73%**	80%	74%	70%	72%
**Ethnicity (%)**	**White**	**77%**	60%	61%	63%	67%	**64%**	60%	61%	63%	67%
	**A.A**	**22%**	7%	3%	30%	30%	**23%**	7%	3%	30%	30%
	**Asian**	**0%**	7%	3%	1%	0%	**2%**	7%	3%	1%	0%
	**N.H./P.I.**	**0%**	0%	0%	1%	0%	**0%**	0%	0%	1%	0%
	**Hispanic**	**1%**	20%	34%	4%	2%	**10%**	20%	34%	4%	2%
	**A.I.**	**0%**	0%	0%	0%	0%	**0%**	0%	0%	0%	0%
	**Mixed**	**0%**	0%	0%	0%	0%	**0%**	0%	0%	0%	0%
	**N.R**	**0%**	2%	0%	1%	0%	**1%**	2%	0%	1%	0%
**Season**		**2015**					**2016**				
		**Total**	18–34*	**35–49**	**50–64**	**65–85**	**Total**	18–34*	**35–49**	**50–64**	**65–85**
**Subject (#)**		**270**	**49**	**40**	**84**	**97**	**270**				
**Gender (%)**	**Male**	**25%**	20%	18%	27%	28%	**29%**	22%	27%	33%	28%
	**Female**	**75%**	78%	73%	68%	81%	**71%**	78%	73%	67%	72%
**Ethnicity (%)**	**White**	**70%**	82%	70%	70%	64%	**66%**	71%	70%	63%	72%
	**A.A**	**20%**	6%	3%	26%	29%	**19%**	12%	5%	22%	26%
	**Asian**	**1%**	0%	8%	1%	0%	**1%**	7%	0%	1%	0%
	**N.H./P.I.**	**0%**	0%	0%	1%	0%	**0%**	0%	0%	0%	1%
	**Hispanic**	**7%**	12%	15%	4%	4%	**6%**	10%	22%	3%	2%
	**A.I.**	**0%**	0	0%	0	0%	**0%**	0%	0%	0%	0%
	**Mixed**	**0%**	0	0%	0	0%	**2%**	0%	0%	3%	1%
	**N.R**	**1%**	0	5%	0	1%	**1%**	0%	2%	0%	1%

*Years of age (y.o.)

**Table 2:** Fluzone vaccine strains.

Year	Influenza Season	Fluzone	Route	IAV (H1N1)	(IAV) H3N2	IBV (Victoria)	IBV (Yamagata)
**1**	**2013**	**SD/HD**	**I.M/I.D.**	**A/California/07/2009**	**A/Texas/50/2012**	**N/A**	**B/Massachusetts/2/2012**
**2**	**2014**	**SD/HD**	**I.M**	**A/California/07/2009**	**A/Texas/50/2012**	**N/A**	**B/Massachusetts/2/2012**
**3**	**2015**	**SD/HD**	**I.M**	**A/California/07/2009**	**A/Switzerland/9715293/2013**	B/Brisbane/60/2008*	**B/Phuket/3073/2013**
**4**	**2016**	**SD/HD**	**I.M**	**A/California/07/2009**	**A/Hong Kong/4801/2014**	**B/Brisbane/60/2008**	B/Phuket/3073/2013*

*QIV was used in Standard Dose Vaccine and TIV was used for High Dose Vaccine.

In 2015–2016 and 2016–2017 seasons, the Yamagata stains changed and the Victoria strains were included for a QIV formulation.

### Hemagglutinination-inhibition (HAI) assay

The hemagglutination inhibition (HAI) assay was used to assess functional antibodies to the HA able to inhibit agglutination of turkey erythrocytes. The protocols were adapted from the WHO laboratory influenza surveillance manual [10] WHO Manual. To inactivate non-specific inhibitors, sera were treated with receptor-destroying enzyme (RDE) (Denka Seiken, Co., Japan) prior to being tested. Briefly, three parts of RDE was added to one part of sera and incubated overnight at 37C. RDE was inactivated by incubation at 56C for 30–45 min and then cooled to room temperature before diluting with 1x PBS or 0.85% NaCl to a final sera concentration of 1:10. RDE-treated sera was serially diluted in PBS two-fold across v-bottom microtiter plates. An equal volume of each influenza virus (25 μl), adjusted to a concentration of ~8 hemagglutination units (HAU)/50 μl, was added to each well. The plates were covered and incubated at room temperature for 20 min, and then 0.8% turkey erythrocytes (Lampire Biologicals, Pipersville, PA, USA) in PBS were added. Red blood cells were stored at 4C and used within 72 h of preparation. The plates were mixed by agitation and covered, and the RBCs settled for 30 minutes at room temperature. The HAI titer was determined by the reciprocal dilution of the last well that contained non-agglutinated RBCs. Positive and negative serum controls were included for each plate. Seroprotection was defined as HAI titer ≥1:40 and seroconversion as a 4-fold increase in titer compared to baseline resulting in a titer of ≥1:40, as per the WHO and European Committee for Medicinal Products to evaluate influenza vaccines [11]. People were considered seronegative with a titer less than 1:40.

### Anti-HA enzyme linked immunosorbent assay (ELISA)

ELISA was performed to assess the presence of total HA-reactive antibodies. Immulon 4HBX plates (Thermo Fisher, Waltham, MA, USA) were coated overnight at 4°C with 500 ng/ml of recombinant HA (rHA) in carbonate buffer, pH 9.4, containing 5 micrograms/ml fraction V bovine serum albumin (BSA) (Equitech-Bio, Kerrville, TX, USA) (50 microliters/well) in a humidified chamber. Plates were coated with full length rHA proteins: A/Texas/50/2012, A/Switzerland/9715293/2013, or A/California/07/2009 (Protein Sciences Corp, Meriden CT USA). Plates were blocked with blocking buffer (PBS containing 5% BSA, 2% bovine gelatin, and 0.05% Tween 20) for 60 min at 37C. Serum samples were serially diluted 3-fold in blocking buffer, and plates were incubated with serum overnight at 4C. Human sera samples were freshly prepared in Blocking Buffer at a 1:500 dilution. Plates were washed four times with 1x PBS, and were then incubated with 2° antibody Goat Ant-human IgG Fc-HRP (Southern Biotech, Birmingham AL USA) at a 1:4000 dilution in blocking buffer for 90 mins at 37C. Secondary antibody was washed four times with 1x PBS following incubation, ABTS Diammonium Salt (aMReSCO, Solon, Ohio, USA) working solution was then aliquoted into wells and incubated for 20 mins. Plates were read using a spectrophometer (BioTek, Winooski, VT, USA) at 414 nm (OD_414_). End-point titers are reported as the Log_10_ of the reciprocal serum dilution at which the OD_414_. The average of the background values was determined plus three times standard deviation was subtracted from the end-point dilution titer to achieve the final value. Positive and negative controls were run on each plate.

### Viruses and HA antigens

Influenza viruses were obtained through the Influenza Reagents Resource (IRR), BEI Resources, the Centers for Disease Control (CDC), or were provided by Sanofi Pasteur. Viruses were passaged once in the same growth conditions as they were received, in 12-day old embryonated chicken eggs or semi-confluent Madin-Darby canine kidney (MDCK) cell culture (as per the instructions provided the World Health Organization [10]. Virus lots were titrated with turkey erythrocytes and made into aliquots for single-use applications. The H1N1 virus panel includes A/South Carolina/1/1918 (SC/18), A/Puerto Rico/8/1934 (PR/34), A/Weiss/JY2/1943 (Weiss/1943), A/Fort Monmouth/1/1947 (FM/47), A/Denver/1/1957 (Den/57), A/New Jersey/6/1976 (NJ/76), A/USSR/90/1977 (USSR/77), A/Brazil/1/1978 (Braz/78), A/Chile/1/1983 (Chile/83), A/Singapore/6/1986 (Sing/86), A/Texas/36/1991 (TX/91), A/Beijing/262/1995 (Bei/95), A/New Caledonia/20/1999 (NC/99) A/Solomon Island/3/2006 (SI/06), A/Brisbane/59/2007 (Bris/07), A/California/07/2009 (CA/09), A/Michigan/45/2015 (Mich/15). The H3N2 vaccine panel includes the following strains: A/Hong Kong/1/1968 (HK/68) (NR-28620), A/Port Chalmers/1/1973 (PC/73), A/Mississippi/1/1985 x PR/8 (Miss/85) (NR-3502), A/Sichuan/60/1989 x PR/8 (Sic/89) (NR-3492), A/Nanching/933/1995 (Nan/95), A/Sydney/05/1997 (Syd/97), A/Panama/2007/1999 (Pan/99), A/Fujian/411/2002 (Fuj/02), A/New York/55/2004 (NY/04) (FR-462), A/Wisconsin/67/2005 (Wisc/05) (FR-397), A/Brisbane/10/2007 (Bris/07), A/Perth/16/2009 (Per/09), A/Victoria/361/2011 (Vic/11), A/Texas/50/2012 (TX/12), and A/Switzerland/9715293/2013 (J1506B) (Switz/13), and A/Hong Kong/4801/2014 (HK/14). All viruses were characterized for ability to agglutinate turkey erythrocytes. All viruses were propagated in embryonated chicken eggs.

### Statistical methods

The statistical significance of the differences was calculated by two-tailed paired Student t-test with Wilcoxon-sign rank test comparing day 0 to day 21. Values were considered significant for p ≤ 0.01. Unless otherwise stated, data from at least three independent experiments.

## Results

### Demographics of volunteers

Demographic characteristics of the volunteers showed that in the 2013 season, of the 127 eligible volunteers who enrolled ~75% of the study participants were between the ages of 50–85 y.o. (Table 1). For each of the next three seasons, approximately twice as many people were enrolled. In the 2014 season, 16% of the 276 eligible volunteers who enrolled were between the ages of 18–34 years old (y.o) (young age group) and 14% were 35–49 y.o. (adult age group) (Table 1). Approximately twice as many enrollees were over the age of 50 with 69% of the subjects between 50–85 years of age (36% between 50–64 y.o. (middle age group) and 33% between 65–85 y.o. (elderly age group). A similar distribution of subjects in each age group was generally maintained over the 4 years of the study (Table 1). There were ~3 times more women enrolled in the study than men and 64–70% of the volunteers were self-identified as White, with 20–25% classified as African American and 7–10% self-identified as Hispanic/Latino (Table 1). Of the 275 volunteers enrolled in 2014, 180 were re-enrolled in the study in the next three seasons and 220 participated in the next two seasons (data not shown). In all four seasons, the H1N1 component of the vaccine remained unchanged (Table 2), however, the H3N2 component was changed twice in years 3 and 4 of the study. The IBV components were the same in years 1 and 2 and changed for years 3 and 4.

### Hemagglutination-inhibition (HAI) response to the vaccine strains

**Fig 1:**
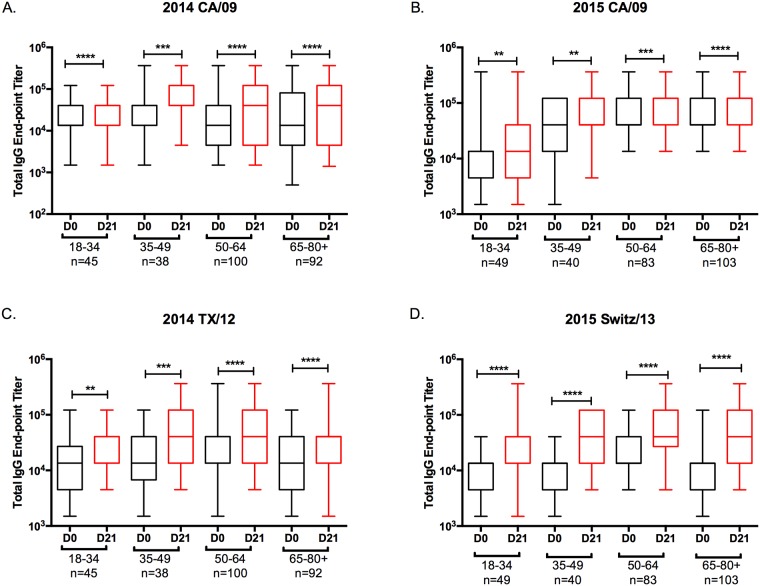
ELISA titers. The box-and-whisker plots show the lower (Q1) and upper (Q4) quartile representing the IgG endpoint dilution titer for anti-HA antibodies. The diagram also shows the geometric mean titer (GMT) for day 0 and day 21 post-vaccination for each age group. The n value per age group is listed on the x-axis. (A) Serum samples were tested (A) 2014 season against rHA for the H1N1 component of the vaccine, A/California/07/2009 (CA/09). (B) 2015 season against rHA for the H1N1 component of the vaccine, A/California/07/2009 (CA/09). (C) 2014 season against rHA for the H3N2 component of the vaccine, A/Texas/50/2012 (TX/12). (D) 2014 season against rHA for the H3N2 component of the vaccine, A/Switzerland/9715293/2013 (Switz/13). *p≤0.05; *p≤0.01; *p≤0.001; *p≤0.0001.

**Fig 2:**
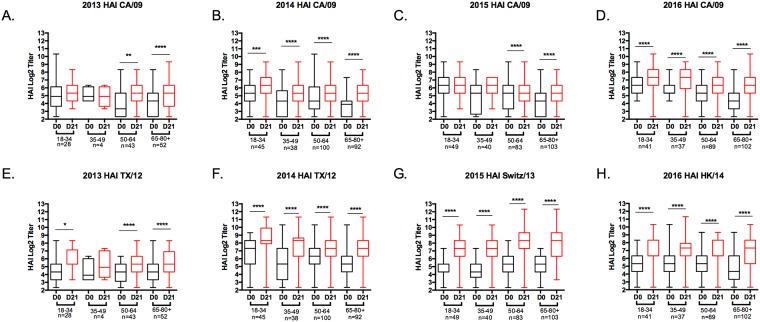
Hemagglutination inhibition (HAI) activity in serum antibody induced by Fluzone^™^. HAI titers were determined from pre- and post-vaccination serum samples against H1N1 (A-D) and H3N2 (E-H). Values of each individual titer are the geometric mean titers plus standard errors of the means (SEM) (error bars). The box-and-whisker plots show the lower (Q1) and upper (Q4) quartile representing the IgG endpoint dilution titer for anti-HA antibodies. The diagram also shows the GMT for day 0 and day 21 post-vaccination for each age group against H1N1 (A-D) and H3N2 (E-H) components of the vaccine. The n value per age group is listed on the x-axis. Serum samples were tested (A and E) 2013 season, (B and F) 2014 season, (C and G) 2015 season, (D and H) 2016 season. *p≤0.05; *p≤0.01; *p≤0.001; *p≤0.0001.

**Fig 3:**
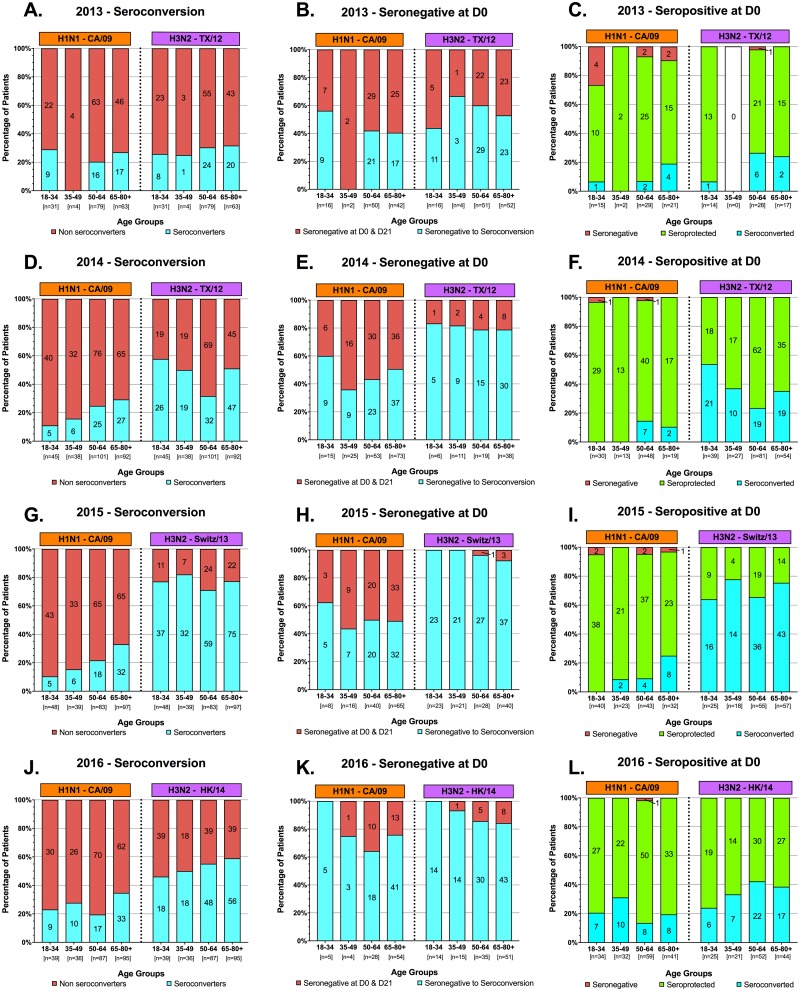
Seroconversion vs. seroprotection. Stacked bars graphs represent the seroconversion to the H1N1 and H3N2 components. The number of individuals for each age group per season are listed on the x-axis. For panels A, D, G, J, subjects were determined to be seroconverters following a 4-fold rise in HAI titer with a titer >1:40 at day 21 (blue) or non-seroconverters (red). Subjects seronegative (<1:40) at day 0 prior to vaccination (B, E, H, K) that seroconverted (four-fold rise in titer with a titer of >1:40) at day 21 are highlighted in blue and those subjects that remained seronegative (<1:40) at both day 0 and 21 are highlighted in red. Subjects that were seropositive (>1:40) at day 0 prior to vaccination (C, F, I, L) that seroconverted (four-fold rise in titer with a titer of >1:40) at day 21 are highlighted in blue and those subjects that dropped in titer to a seronegative state (<1:40) are highlighted in red. Subjects that were seropositive at day 0 and remained seropositive at day 21 are highlighted in green.

In 2014 and 2015, for nearly all age groups there was a statistically significant increase in the total anti-HA IgG antibody titer against the vaccine strains in response to vaccination (Fig 1). For H1N1, a statistically significant increase in the HAI-mediating antibodies elicited by the vaccine was found in the elderly age group in each of the four seasons (Fig 2). Increases in HAI activity was also observed in the other three younger age groups, but there was variability from one year to the next. In contrast, for H3N2, in almost all age groups, there was a statistically significant increase in HAI specific antibodies (Fig 2E–2H). In almost every age group and in each season, the HAI geometric mean titer (GMT) to the vaccine strains increased following vaccination (Table 3) with the fold-increase of the response trending higher in the older age groups. Few people had seroprotective titers (0–35%) to the H1N1 component of the vaccine in any season (Fig 3A, 3D, 3G and 3J) with the highest number of non-seroconverters in the young, 18–34 y.o. group. In contrast, the number of people seroconverting to the H3N2 component of the vaccine was significantly higher (23–82%).

**Table 3:** Hemagglutinin-inhibition (HAI) GMT.

H1N1 CA/09	GMT +/- SEM			H3N2	GMT +/- SEM		
Age (y.o.)				Age (y.o.)			
2013	D0	D21	Fold Rise	TX/12 2013	D0	D21	Fold Rise
**18–34**	29 +/- 226*	45 +/- 90	1.75	**18–34**	29 +/- 58	52 +/- 84	2.42
**35–49**	34 +/- 28	28 +/- 31	0.82	**35–49**	20 +/- 13	34 +/- 10	1.7
**50–64**	19 +/- 64	39 +/- 93	2.14	**50–64**	35 +/- 88	81 +/- 318	2.41
**65–85**	16 +/- 38	38 +/- 188	2.24	**65–85**	22 +/- 162	52 +/- 106	2.2
**2014**				**TX/12 2014**			
**18–34**	41 +/- 73	63 +/- 105	1.54	**18–34**	109 +/- 178	416 +/- 587	3.82
**35–49**	20 +/- 61	38 +/- 107	1.9	**35–49**	48 +/- 232	210 +/- 471	4.38
**50–64**	24 +/- 139	50 +/- 113	2.04	**50–64**	65 +/- 161	148 +/- 398	2.28
**65–85**	14 +/- 33	35 +/- 67	2.5	**65–85**	39 +/- 162	129 +/- 364	3.31
**2015**				**Switz/13 2015**			
**18–34**	71 +/- 159	86 +/- 130	1.21	**18–34**	25 +/- 30	170 +/- 217	6.80
**35–49**	32 +/- 90	57 +/- 56	1.78	**35–49**	23 +/- 30	184 +/- 334	8.00
**50–64**	28 +/- 101	55 +/- 119	1.89	**50–64**	41 +/- 62	255 +/- 1001	6.2
**65–85**	16 +/- 57	39 +/- 79	2.43	**65–85**	30 +/- 37	230 +/- 850	7.67
**2016**				**HK/14 2016**			
**18–34**	83 +/- 126	172 +/- 251	2.07	**18–34**	44 +/- 78	152 +/- 301	3.45
**35–49**	62 +/- 83	125 +/- 154	2.02	**35–49**	39 +/- 212	125 +/- 457	3.21
**50–64**	43 +/- 85	83 +/- 131	2.33	**50–64**	36 +/- 85	122 +/- 206	4.78
**65–85**	25 +/- 61	73 +/- 158	2.48	**65–85**	30 +/- 131	153 +/- 306	5.56

*GMT +/- S.E.M.

Prior to vaccination, antisera were scored as seronegative (defined as an HAI titer less than 1:40) (Fig 3B, 3E, 3H and 3K) or seropositive (defined as an HAI titer greater than 1:40) (Fig 3C, 3F, 3I and 3L) against both the H1N1 and H3N2 vaccine components. Following vaccination at day 21, sera from individuals that were seronegative at day 0 were assayed for seroconversion to the H1N1 and H3N2 strains in the vaccine (Fig 3B, 3E, 3H and 3K). Young volunteers had higher seroconversion rates (56–100%) against the H1N1 component following vaccination compared to the other three age groups (36–76%), even though the n for the young volunteers in this category are quite low. While there was no statistical difference in any season, 90–100% of the seronegative volunteers, regardless of age, seroconverted to the H3N2 component by day 21 in the 2015 and 2016 season (Fig 3H and 3K) and 44–83% in 2013 and 2014 (Fig 3B and 3E). In contrast, the number of seropositive volunteers at day 0 that seroconverted to the vaccine strains by day 21 was quite low, particularly for the H1N1 component (0–31%). For the H3N2 component, the seroconversion rates were low for all seasons, except 2015 (Fig 3I). The majority (up to 90%) of these volunteers remained seropositive to the vaccine strains without a 4-fold rise in HAI titers (Fig 3C, 3F, 3I and 3L, stacked green bars). A small number of these volunteers had a decline in HAI titers and became seronegative. Overall, the rates of seroconversion are dependent on the HAI titers to the vaccine component prior to vaccination with higher seroconversion rates in people that are documented to be seronegative to the vaccine component prior to vaccination.

### Seroprotective HAI titers analyzed by age group over multiple seasons

**Fig 4:**
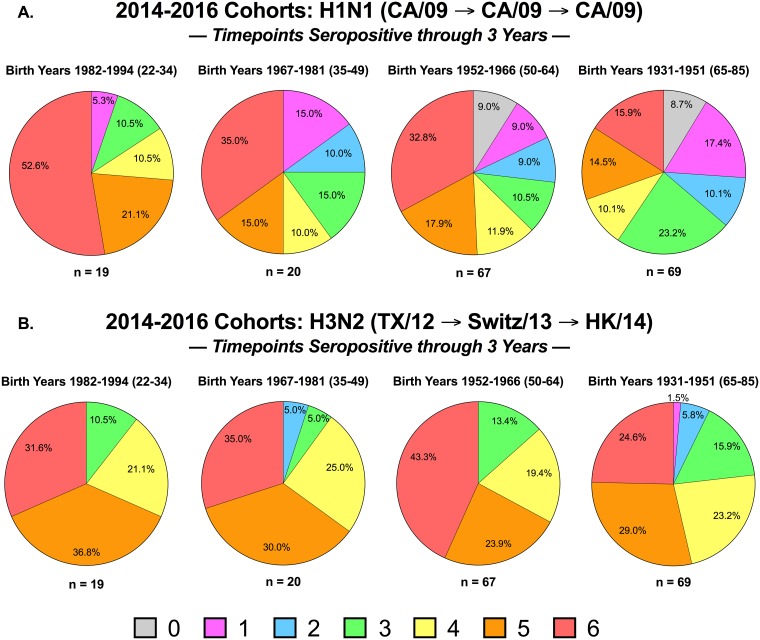
Seropositivity over multiple influenza seasons. Subjects were immunized over 3 consecutive seasons, 2014, 2015, and 2016. Each subject was assessed for seropositive titers to the H1N1 and H3N2 components of the vaccine at day 0 and day 21 immunized were categorized according to their seroprotection status at each of the 6 time points analyzed (*i*.*e*., days 0 and 21 in all 3 years). Subjects in each age group that had HAI titers less than 1:40 at all 6 time points were assigned a value of “0”. Subjects with an HAI titer of 1:40 or greater at one of the 6 time points was categorized as a “1”. Subjects with an HAI titer of 1:40 or greater at two of the 6 times was categorized as a “2”, at three of the 6 time points, was categorized as a “3”, at four of the 6 times was categorized as a “4”, at five of the 6 times was categorized as a “5”, and subjects with was greater than 1:40 all 6 times was categorized as a “6”. Pie charts for each of these 7 categories was assessed for each age group against the H1N1 or H3N2 component. The n value for each age group was listed beneath each pie chart.

In order to determine the effect of pre-existing antibodies with HAI activity to current vaccine strains, we categorized the HAI responses to the H1 and H3 vaccine strains over three consecutive northern hemisphere seasons (*i*.*e*. 2014-2015-2016) (Fig 4). Subjects immunized over 3 consecutive seasons were categorized according to their seroprotection status at each of the 6 time points analyzed (*i*.*e*., days 0 and 21 in all 3 years). For example, individuals in each age group that had HAI titers less than 1:40 at all 6 time points were assigned a value of “0”. If an individual had an HAI titer of 1:40 or greater at one of the 6 time points, their response was categorized as a “1”. If an individual had an HAI titer of 1:40 or greater at two of the 6 times, this response was categorized as a “2”, at three of the 6 time points, this response was categorized as a “3”, at four of the 6 times, this response was categorized as a “4”, at five of the 6 times, this response was categorized as a “5”, and if the titer was greater than 1:40 all 6 times, this response was categorized as a “6”.

One hundred and seventy-five individuals were enrolled and vaccinated in the 2014-2015-2016 influenza seasons. There were ~3.6 fold more “repeaters” in the two oldest age groups than in the two youngest age groups. For volunteers in the youngest cohort, 53% of them had sera with HAI activity greater than 1:40 against H1N1 component of the vaccine at all 6 time points and were categorized as a “6” (Fig 4A) and another 21% of the volunteers were in category 5. Therefore, 74% of the young volunteers (22–34 y.o.) were in categories 5 or 6 against the H1N1 component, CA/09 (Fig 4A). The percentage of volunteers in these categories decreased in older age groups with only 31% of volunteers placed in category 5 and 6 in the elderly age group (65–85 y.o). There was an increase in the number volunteers in the two oldest age groups in category “0” (~9%) with no HAI activity against CA/09 H1N1 at any time point. In the volunteers under the age of 50, no one was in categories 0 or 1 for the H3N2 component (Fig 4B). For example, there were 69% of the young volunteers (18–34 y.o.) having HAI activity against the HK/14 vaccine strain at 5 or 6 time points (Fig 4B), whereas only 54% of the elderly volunteers had this phenotype. For both H1 and H3 vaccine strains, individuals in the two oldest age groups had HAI activity that represented all 7 of the HAI categories. In contrast, the two youngest age groups had individuals with HAI activity that represented the top 4–5 categories (Fig 4).

### HAI activity against a panel of historical vaccine H1N1 and H3N2 influenza A strains

**Fig 5:**
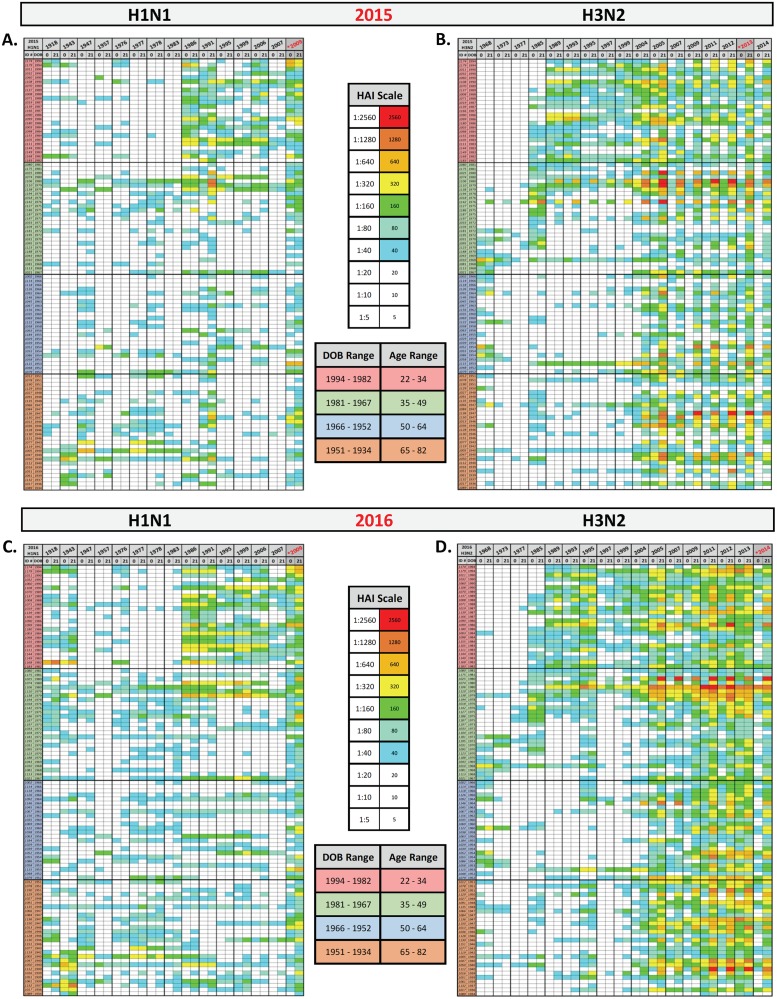
Heat Map of HAI results. Serum samples were collected from subjects at day 0 and day 21 post-vaccination and HAI activity of each serum samples was tested against a panel of H1N1 and H3N2 historical influenza viruses. A heat map of each subject’s HAI titer against each strain is depicted for (A) H1N1 2015 season, (B) H3N2 2015 season, (C) H1N1 2016 season, (D) H3N2 2016 season. The list of strains are shown on the upper x-axis in chronological order from left to right, oldest to youngest. The subjects were categorized by each age group (youngest to oldest listed from top to bottom of the heat map) on the y-axis. Those subjects between the ages of 18–34 y.o. are highlighted in red, subject between the ages of 35–49 y.o in green, subject between the ages 50–64 y.o in blue, and 65–85 y.o. in orange. HAI titers greater than 1:40 are highlighted on a color scale and HAI less than 1:40 are highlighted are not colored (1:20, 1:10, and 1:5).

Sera collected prior to vaccination and 21-days post-vaccination from volunteers that participated in three consecutive seasons (2014–2016) were tested for HAI activity against a panel of 32 historical H1N1 and H3N2 isolates representing the time-period between 1918 and 2014 (Fig 5). Volunteers in all age groups, but particularly for those over the age of 40, had HAI activity prior to vaccination against older H1N1 strains in the panel, pre-1957 and the era between 1977–1983, which was more pronounced than younger volunteers (Fig 5A and 5C). Volunteers in the young group generally had sera at day 0 with pre-existing HAI titer to recent H1N1 seasonal strains from the 1986–2006, whereas older volunteers in the cohort had sera with little HAI activity against recent strains, except for the TX/91 H1N1 virus (Fig 5A and 5C). Both younger and older cohorts had pre-existing HAI activity against the TX/91 strain and it was more likely that HAI activity to TX/91 was a direct result of having recalled immunity or ‘back-boosting’ to shared antigenic domains between this virus and the current H1N1 vaccine strain following vaccination. In contrast, the geometric mean HAI titer (GMT) increased in all age groups following vaccination against the H3N2 component of the QIV vaccine in each season (Fig 5B and 5D and Table 3). In addition, there was a back boosting of HAI titers on day 21 post-vaccination to the historical strains, TX/12, Vic/11, Perth/09, Bris/07, and Wisc/05 in all age groups. Prior to vaccination (day 0), volunteers in the youngest age group had antibodies with HAI activity against more viruses in the panel representing historical strains dating back to 1985 with middle aged or older volunteers recognizing fewer of these older strains (Fig 5B and 5D). Overall, the HAI activity to the H1 and H3 vaccine strains each season were statistically similar between seasons regardless of the age group (Fig 5).

### Seroconversion to the vaccine strain enhances back boosting

To examine the effect of age and pre-existing antibodies on the back-boosting phenomenon, HAI activity against a panel of historical vaccine strains was assessed from pre- and post-vaccination sera (Table 4). The number of strains recognized by the antisera with a titer of 1:40 or higher using day 21 (post-vaccination) sera was determined and compared to the number of strains recognized using day 0 (pre-vaccination) sera. The increase or decrease in number of strains recognized at day 21 compared to day 0 was recorded and placed into three categories: increase by 2 or more, decrease by 2 or more, or little (+/- 1) to no change (Table 4). For example, in 2014, 18% of the sera collected at day 21 from the young cohort gained HAI activity against 2 or more H1N1 viruses in the panel following vaccination compared to day 0 (Table 4), whereas 15% of the young volunteers recognized fewer viruses in the panel compared to day 0 (Table 4). In contrast, 42% of the people in the aged cohort had antisera that recognized 2 or more viruses in the panel and only 2% recognized fewer strains at day 21 compared to day 0. Similar results were observed in the 2015 season against the H1N1 strains.

**Table 4:** Percent of individuals with antisera with HAI activity that recognized more, fewer, or similar number of strains in the panel post-vaccination compared to pre-challenge.

	Year-Subtype	≤ 2*	Similar	≥ 2*	Year-Subtype	≤ 2	Similar	≥ 2
Age	2014-H1N1				2014-H3N2			
18–34		18%	67%	15%		13%	87%	0%
35–49		32%	68%	0%		60%	37%	3%
50–64		44%	56%	0%		61%	36%	3%
65–85		42%	55%	2%		59%	40%	1%
	2015-H1N1				2015-H3N2			
18–34		13%	73%	15%		37%	47%	16%
35–49		26%	46%	22%		50%	48%	2%
50–64		37%	55%	8%		61%	34%	5%
65–85		39%	55%	6%		66%	28%	6%
	2016-H1N1				2016-H3N2			
18–34		33%	68%	0%		28%	72%	0%
35–49		59%	38%	3%		44%	56%	0%
50–64		35%	65%	0%		48%	52%	0%
65–85		35%	62%	3%		53%	47%	0%

*≥2 or more = Antisera detects 2 or more viruses post-vaccination compared to pre-vaccination in the HAI assay in the panel

Similar = Antisera detects 1 more, the same, or 1 fewer virus post-vaccination compared to pre-vaccination in the HAI assay in the panel post-vaccination

≤2 or fewer = Antisera detects 2 or fewer viruses post-vaccination compared to pre-vaccination in the HAI assay in the panel

The same sera were tested against the panel of H3N2 viruses and in 2014, the percentage of volunteers per age group that had increased or decreased recognition of H3N2 by age was similar to 2014 H1N1 analysis (Table 4). However, in 2015, the percentage of individuals in the young cohort with HAI activity in their sera that recognized 2 or more H3N2 in the panel almost tripled (13% to 37%), but no change in the other age groups were observed. Interestingly, 16% of the 2015 young cohort had sera that recognized 2 or fewer viruses in the panel, whereas no one in the young cohort recognized 2 or fewer viruses in 2014 and 2016 (Table 4).

### Breadth of ‘back-boosting’ response post-vaccination as a function of influenza exposure history or age

**Fig 6:**
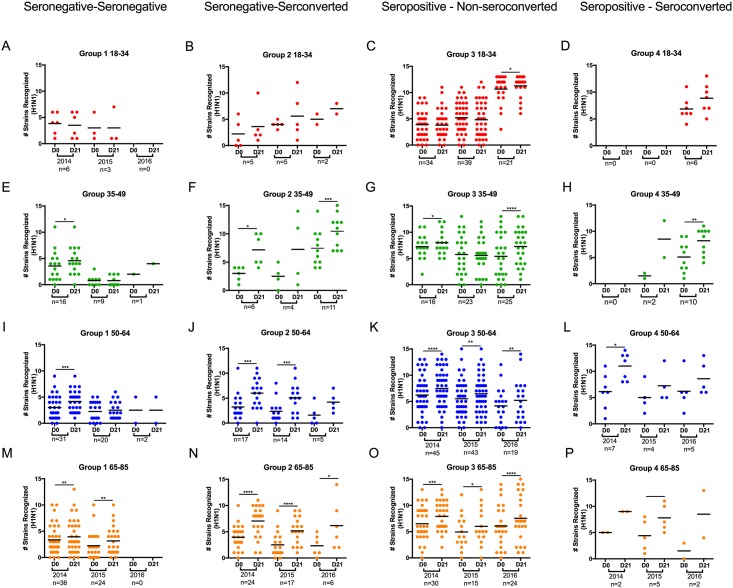
Vaccine induced back boosting to H1N1 historical viruses. HAI activity in sera collected from subjects pre- (day 0) and post- (day 21) vaccination were examined for the number of strains in detected with a titer greater than 1:40 against each strain the H1N1 influenza virus panel. Subjects from three consecutive influenza seasons (2014, 2015, 2016), along with the number of subjects per season (n value) are listed on the x-axis at day 0 and 21 post-vaccination. The number of strains recognized by each subject is listed on the y-axis. Panel A-D represent subjects between ages of 18–34 y.o (red symbols). Panel E-H represent subjects between the ages of 35–49 y.o (green symbols). Panel I-L represent subjects between the ages 50–64 y.o. (blue symbols). Panel M-P represent subjects between the ages of 65–85 y.o. (orange symbols). Subjects seronegative (<1:40) to the H1N1 component in the vaccine at day 0 and remained seronegative post-vaccination (Panels A, E, I, M). Subjects seronegative (<1:40) to the H1N1 component in the vaccine at day 0 but seroconverted (four-fold increase in titer with a titer ≥1:40) post-vaccination (Panels B, F, J, N). Subjects seropositive (≥1:40) to the H1N1 component in the vaccine at day 0 but did not seroconvert post-vaccination (Panels C, G, K, O). Subjects seropositive (≥1:40) to the H1N1 component in the vaccine at day 0 and seroconverted (four-fold rise in titer) post-vaccination (Panels D, H, L, P).

**Fig 7:**
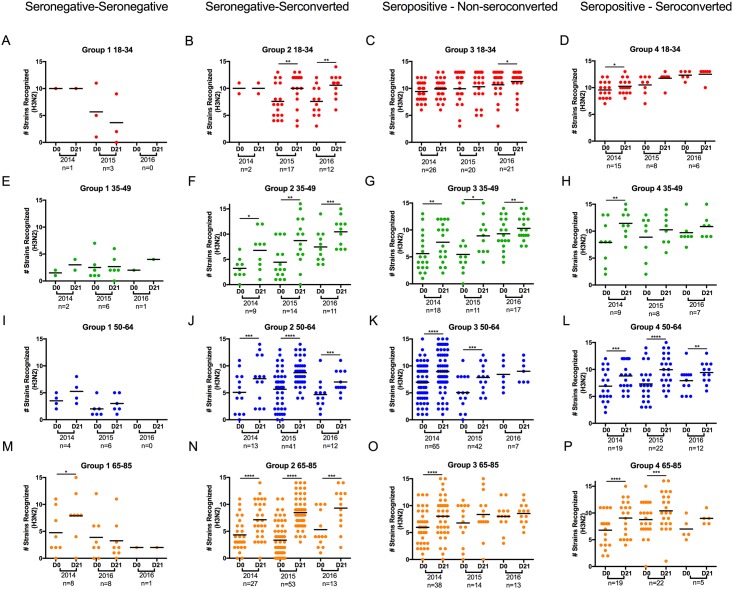
Vaccine induced back boosting to H3N2 historical viruses. HAI activity in sera collected from subjects pre- (day 0) and post- (day 21) vaccination were examined for the number of strains in detected with a titer greater than 1:40 against each strain the H3N2 influenza virus panel. Subjects from three consecutive influenza seasons (2014, 2015, 2016), along with the number of subjects per season (n value) are listed on the x-axis at day 0 and 21 post-vaccination. The number of strains recognized by each subject is listed on the y-axis. Panel A-D represent subjects between ages of 18–34 y.o (red symbols). Panel E-H represent subjects between the ages of 35–49 y.o (green symbols). Panel I-L represent subjects between the ages 50–64 y.o. (blue symbols). Panel M-P represent subjects between the ages of 65–85 y.o. (orange symbols). Subjects seronegative (<1:40) to the H3N2 component in the vaccine at day 0 and remained seronegative post-vaccination (Panels A, E, I, M). Subjects seronegative (<1:40) to the H3N2 component in the vaccine at day 0 but seroconverted (four-fold increase in titer with a titer ≥1:40) post-vaccination (Panels B, F, J, N). Subjects seropositive (≥1:40) to the H3N2 component in the vaccine at day 0 but did not seroconvert post-vaccination (Panels C, G, K, O). Subjects seropositive (≥1:40) to the H3N2 component in the vaccine at day 0 and seroconverted (four-fold rise in titer) post-vaccination (Panels D, H, L, P).

The number of strains in the H1N1 and H3N2 panel recognized by HAI positive immune sera, (pre- and post-vaccination), was then determined as a function of age and classified into 4 different seroconversion groups over the three consecutive seasons (Figs 6 and 7). By way of example, all the young volunteers (total of 93) were classified based upon seroconversion to the vaccine strain. Young volunteers (6 in 2014, 3 in 2015, and 0 in 2016) that had no seroconversion to the H1N1 component of the vaccine were classified as Group 1; seronegative at day 0 and seronegative at day 21 (Fig 6A). On average, these young volunteers had HAI activity against 3–4 of the 17 H1N1 viruses in the panel. Those young age volunteers classified as seronegative at day 0 that seroconverted at day 21 had HAI activity less than 1:40 to the H1N1 vaccine component prior to vaccination, followed by a 4-fold rise in HAI activity to a titer of ≥ 1:40 following vaccination. In 2014, young volunteers in Group 2 (the day 0 seronegative/day 21 seroconverted category) had sera with HAI activity that, on average, recognized 2 of the viruses in the H1N1 panel in 2014 and did not statistically increase post-vaccination at day 21, but there was an increase in the number of historical strains recognized in 2015 and 2016 (Fig 6B). Similar results were observed for those young age volunteers classified as seropositive to the vaccine strain at day 0 that did not seroconvert at day 21, but remained seropositive on day 21 (Fig 6C, Group 3). In fact, people in this age group represented the major phenotype of this age group with the most significant breadth observed. The serum HAI activity of these individuals in Group 3 are similar to those in the seronegative/seroconverted category (Group 2), however, these people did not have a 4-fold increase in HAI activity following vaccination, but they did have a seroprotective titer of 1:40 at day 21 (Fig 6C). No young age volunteers were classified as seropositive on day 0/seroconverted on day 21 (Group 4) in 2014 or 2015 and only 6 were classified in Group 4 in 2016 (Fig 6D). Volunteers in Group 4 had a seroprotective titer (1:40 or greater prior to vaccination) that increased by 4-fold or greater after vaccination on day 21 (Fig 6D). These subjects were categorized as seropositive/seroconverters.

In 2014, 34 of the 45 (75%) young aged volunteers and 82% in 2015 and 67% in 2016 were classified as seropositive/non-seroconverters against the H1N1 vaccine component, however, there was little increase in the average number of viruses in the panel recognized by the sera post-vaccination (Fig 6C). In 2016, a large increase occurred in the number of vaccine strains recognized from an average of 5 in 2015 to an average of 10 strains recognized in 2016. The number of individuals classified as seronegative/seronegative in 2014 increased by age from 13% for the young aged cohort to 39% for the elderly cohort (Fig 6A and 6M) with a concomitant decrease (18% of the total volunteers in this age group) in the number of elderly volunteers classified as seropositive/non-seroconverters (Fig 6C and 6O). However, the number of people in the seronegative/seronegative classification declined in 2015 and again in 2016. While the number of volunteers remained low in seropositive/seroconverted classification in all age groups, there were statistically significant increases in the number of individuals classified as seronegative/seroconverted in the two oldest age groups compared to young age group. In the adult, middle-aged, and elderly age groups, there was a significant increase in the number of H1N1 strains recognized by the sera collected 21 days after vaccination compared to pre-vaccination at day 0 (Fig 6F, 6J and 6N). There was also a significant increase in the number of strains back-boosted in sera collected post-vaccination for those adult, middle-aged, and elderly volunteers classified as seropositive/seroconverted (Fig 6H, 6L, and 6P), as well as those middle-aged and elderly volunteers classified as seropositive/non-seroconverted (Fig 6K and 6O). For the H3N2 component of the vaccine, similar results to the H1N1 distribution and back-boosting of HAI titers were observed in 2014, 2015, and 2016 amongst all age groups with a few outstanding exceptions (Fig 7).

## Discussion

In this study, groups of individuals representing different age groups were vaccinated in four consecutive influenza seasons. This allowed a comprehensive analysis of the effect of repeat vaccination on HAI to be assessed in the same cohort as a function of age. Adult volunteers from 18 to 85 years of age were recruited from one of two sites; Stuart, Florida or Pittsburgh, Pennsylvania. After the initial 2013 season, ~270 individuals were divided into four age classifications to represent young, adult, middle aged and the elderly. There was a mix of ethnicities and ~75% of the volunteers were women. All volunteers in the 2015 and 2016 seasons were vaccinated with the split inactivated (IIV) influenza (standard or high dose) FluZone^™^ vaccine (Sanofi Pasteur). Each vaccine contained two influenza A strains (H1N1 and H3N2) and one or two influenza B strains (Yamagata and Victoria lineages). During the study, the H1N1 component, A/California/07/2009 (CA/09), was constant over the 4 seasons. However, the H3N2 component was changed in 2 out of 4 seasons assessed. In 2013 and 2014, volunteers were vaccinated with the split, inactivated IIV vaccine that contained two influenza A strains and only one influenza B strain (B/Massachusetts/2/2012, Yamagata lineage), which was replaced in 2015 and 2016 with the B/Phuket/3073/2013 (Table 2). For the Victoria lineage strain, B/Brisbane/60/2008, was added to the vaccine in 2015.

Following vaccination, all age groups had a rise in anti-HA IgG antibody titers. However, people in the three oldest age groups were more likely to have increases in HAI titers to vaccine strains at day 21 post-vaccination than people in the 18–34 year old age group. This resulted in higher seroconversion rates in the elderly age group compared to the young age group. This would appear contrary to the yearly reported seroconversion rates published by the U.S. Centers for Diseases Control and Prevention and the World Health Organization each year [12]. The reported average seroconversion rate (defined as a four-fold rise in HAI titer post-vaccination from the pre-vaccination titer) ranges from 50–75% of vaccinated individuals between the ages of 18–49 years of age and 10–30% for the elderly (defined as older than 65 years of age). While discrepant, waning immunity in the elderly season by season might explain the findings in our study. Even though fewer people had a 4-fold rise in HAI titers post-vaccination, in general, the geometric mean HAI titer was higher at day 21 in the young age group and decreased by age, also a finding consistent in the elderly, in particular, that requires annual boosts. However, the fold rise was similar between age groups each season, indicating that the overall elicitation of the HAI response was equally effective in all age groups for both the H1N1 and H3N2 components of the vaccine in all seasons. The response to the influenza B components and comparisons between TIV and QIV vaccination will be reported elsewhere. While the fold rise in HAI GMT was more varied from season to season against the H3N2 component compared to the H1N1 component, the rise between age groups was similar in each season.

Influenza vaccination is less effective in the elderly compared to young adults, in part due to decreased generation of specific serum antibodies [13] [14] [15] [16] [17] [18] switched memory B cells [19] [13] [14] [15] [17] [18], and the presence of long-lived plasma cells (PC) [20] [21] [22]. Moreover, antibody titers generated in response to a booster vaccination depend on B cell stimulation, differentiation to B memory cells generated during the primary response, and stimulation and differentiation of these to make new plasmablasts and PC [23]. Intrinsic B cell defects increase with age and contribute to sub-optimal vaccine responses in humans [18] [17].

In the present study, generally all age groups were not necessarily more likely to seroconvert to the H1N1 component of the vaccine compared to the H3N2 within a given season (Figs 2 or 3). These results did not seem to be influenced by administration of high dose vaccine in the elderly compared to elderly that received standard dose (data not shown). However, when individuals were categorized at day 0 as seronegative (HAI titer less than 1:40), the seroconversion rates were similar across all age groups in all 4 seasons with the caveat that there were considerably fewer individuals in the young, 18–34 y.o., age group. Similar seroconversion results were observed when the people were seropositive at day 0 (HAI titer greater than 1:40). The vast-majority of individuals that were seropositive did not increase their HAI titer by 4-fold following vaccination, particularly to the H1N1 component of the vaccine. These results indicate that people from all age groups can seroconvert to the influenza A components of the vaccine following vaccination, regardless if the individual is seropositive or seronegative prior to vaccination. However, the number of people in each category varies by age group, with youngest individuals (18–34 y.o.) primarily in a seropositive state and therefore, remain seropositive without seroconverting post-vaccination. This may be due to younger people having more durable antibody responses than the elderly (Fig 4). Individuals, regardless of age, are more likely to seroconvert when they are seronegative prior to vaccination, however, there are more elderly people that are seronegative prior to vaccination and therefore, have more people that have the potential to seroconvert to the vaccine.

When we examined the ability to maintain a seropositive HAI titer over consecutive seasons, younger people were more likely to be seropositive in consecutive seasons than elderly individuals (Fig 4). The duration and quality of anti-influenza antibodies and the generation of immunological memory to vaccines is critical for protective immunity. The antibodies made during a recall memory response differ from those generated during a primary response, as the class/isotype of the secreted antibody is usually switched [24] [25]. Moreover, antibodies appear in serum faster and the affinity of the antibodies generated is higher than in the primary response. Therefore, the ability to maintain seroprotective HAI titers against the influenza viruses is critical for protection. The elderly population consists of more individuals that are seronegative and therefore, those that do seroconvert are more likely to have their HAI titer wane over consecutive seasons, even when receiving the same split, inactivated IIV each season.

In addition to examining the effects of age on influenza vaccination, the effect of pre-existing anti-influenza immunity was examined on vaccine induced recall or back-boosting of serum antibody response to past historical vaccine strains (Fig 5). HAI titers were examined pre- and post-vaccination against a set of H1N1 and H3N2 influenza A viruses representing past vaccine strains. Young people (18–34 y.o.) were more likely to have pre-existing HAI antibodies against the strains from 1986 to 2009 compared to individuals in the other three age groups. Older individuals, particularly those in the 35–49 y.o. adult age group had higher pre-existing HAI antibodies against H1N1 strains from 1977–1983. Almost none of the individuals in the youngest age group had pre-existing antibodies to strains from this era. In contrast, young individuals recognized almost all of the H3N2 strains from 1985 to present, whereas older individuals had a more restricted HAI activity against strains from 2004 to present. Interestingly, in all age groups, there was an increase, or “back-boost”, in HAI titer targeting historical strains at day 21 across all age groups. Back boosting of HAI titers to past strains was reported by Fonville *et al*. [26] and is dependent on the pre-exposure history and more recently has been termed ‘Immunological imprinting’ [27]. The magnitude of the response to historical strains in general declines with increasing antigenic distance from the strain used in the vaccine. The long-term persistence of increased antibody titers was more specific to the antigenic era of the likely infecting strain, but still spanned multiple antigenic clusters. The intensity of the ‘back-boosting’ response was related to how closely the vaccine strain matched the antigenic cluster of those target viruses evaluated in the panel. This phenomenon was observed more often with the H3N2 viruses than with the H1N1 viruses. The HA from the pandemic H1N1 viruses is not antigenically similar to the human seasonal viruses that preceded its emergence in 2009 [28] [29] [30]. All age groups recognized a similar number of H1N1 historical strains, but these strains differed per age group, as might be expected based on different immunological imprinting.

We did observe an age-related effect in the number of back-boosted strains recognized post-vaccination at day 21 (Table 3). Younger individuals were less likely to increase the number of back boosted strains recognized following vaccination compared to individuals over the age of 35 years of age. In fact, a small percentage of young people were more likely to have a decline in the number of back-boosted strains with little or no individuals in the other age groups showing a decline in the number of strains recognized. Older people were more likely to have an increase in the number of back-boosted strains recognized post-vaccination. This finding is consistent with back-boosting being associated with seroconversion and likely depth of exposure history. As a group, young people have less seroconversion because they are already seropositive to the vaccine strain prior to vaccination each season.

To explore the reason for these back-boosting responses, we categorized the individuals in each age group as either seronegative or seropositive on day 0 in the three consecutive seasons and further sub-categorized individuals based on whether or not they seroconverted to the vaccine strain. Then, we identified the number of strains in the historical panel by subtype that each individual recognized pre- and post-vaccination. For H1N1 (Fig 6) in all three seasons, most of the seronegative individuals are elderly, whereas there are relatively few people in the youngest age group who are seronegative prior to vaccination. In the middle-age (50–64 y.o.) and elderly groups (65–85 y.o.) that were seronegative at day 0, ~50% of the people seroconverted following vaccination (seronegative/seroconverted). The largest proportion of people are seropositive at day 0 and do not seroconvert at day 21, these individuals do remain seropositive (seropositive/non-seroconverted), across all age groups, although in general, more people in the adult (35–49 y.o), middle age (50–64 y.o.) and elderly (64–85 y.o.) populations. However, a small subset of the seropositive individuals do seroconvert (seropositive/seroconverted). It is not clear, but the increased numbers of people in the two youngest age groups that are seropositive at day 0 to the H1N1 component of the vaccine in 2016, and that do seroconvert at day 21, may be due to repeated vaccination or subclinical infections [31] over consecutive seasons. Serial vaccination may be associated with reduced influenza vaccine performance against H3N2 epidemics between 2010 and 2014 [32]. It was speculated that the hierarchy of HAI antibody responses against historical influenza strains are shaped by original priming, or imprinting, as well as back-boosting immune responses [33–37]. Annually-repeated vaccination may have negative effects on the immune response by accelerating antibody re-focusing toward prior, rather than evolved epitopes, or for selection of cross-reactive, non-neutralizing antibody responses [38].

In this study, there is also little evidence supporting the hypothesis that repeated annual vaccination limits a vaccinee’s ability to mount breadth of immunity against past strains. Based upon the data presented here, we hypothesize that repeated vaccination pushes all individuals into a consistently seropositive state as indicated in Table 3. This phenomenon is most readily observed in the young, but does occur across all age groups. Therefore, the lower seroconversion rates observed against the H1N1 component of the vaccine is artificially low because most of the young individuals enter the study in a seropositive state and maintain that status for the duration of the study (Table 4). Annual vaccination formulation changes do result in broader antibody results [39] and regulatory agencies may consider re-formulating the various vaccine components annually.

With respect to the H3N2 component of the vaccine, young people recognize a high number of historical strains on day 0 (Fig 7), these HAI data are also represented on an individual level as a heat map (Fig 5B). Unlike the responses to H1N1, few people were seronegative, either pre- or post-vaccination in any of the age groups. Whether this was a result of annual updating of the H3N2 component of the vaccine in each of the seasons represented is not clear, unlike the H1N1 component which remained constant. Although the strains used in the vaccine changed multiple times over the duration of the study, antibodies elicited against the vaccine strains tended to be cross-reactive against unmatched strains in the HAI assay [40]; an observation confirmed through antibody repertoire profiling of individuals exhibiting breadth of immunity and that will be reported elsewhere. Similar to what was observed with H1N1, nearly all of the volunteers that were seronegative prior to vaccination against the H3N2 component seroconverted to the vaccine by day 21. Seroconversion was associated with a rise in the number of back-boosted strains recognized by the elicited antisera collected from the volunteers regardless of age. For individuals that were seropositive prior to vaccination, there was a more modest back-boosting to the panel of H3N2 strains. The increase in the number of back boosted strains was more pronounced in the older groups and was less obvious in the young age group. Seropositive young individuals had antisera that recognized 10 or more of the 15 historical strains tested prior to vaccination and therefore, even if there was a rise in the number of back boosted strains recognized post-vaccination, the boost was less significant (Fig 7C and 7D).

Overall, the back-boosting to past strains was associated with seroconversion to the vaccine strain in each season. A person’s pre-existing immunity to influenza attenuates influenza infection by subsequently strengthening the influenza vaccine induced responses [41]. Influenza infection, and possibly vaccination, imprints an immunological memory response that is boosted following influenza vaccination and that results in the elicitation of antibodies that recognize multiple influenza viral variants [41]. Influenza vaccine antibody responses are shaped by pre-existing antibody responses to influenza [22, 42–44]. Recently, it was demonstrated that ~60% of the serum antibody response post-vaccination to influenza is due to the boosting effect of pre-existing antibody clonotypes [45] and not *de-novo* responses to the vaccine. Therefore, a higher pre-vaccination antibody titer to a specific influenza virus strain results in a more dominant boost effect of pre-existing antibodies and the emergence of fewer *de novo* vaccine-elicited antibodies [45]. In the present study, younger and older individuals enter each season with different breadth of pre-existing antibodies prior to vaccination. When the young and older volunteers are divided into similar seroconversion/seronegative categories based on their serological status (seropositive/seronegative), they appear to react in a similar manner to vaccination. But the proportion of volunteers assigned to these categories differ by age with almost all the young people in the seropositive category prior to vaccination and fewer elderly in this category. Therefore, a major difference between the young and elderly age groups is the number of people represented in these age groups and how they parse out into the various phenotypes (or categories). People in the elderly age group (65–85 y.o.), taken as a whole, may require more frequent vaccination as a result of having a less robust antibody response to influenza vaccination. Regardless, when considering responses on an individual basis, once people are phenotyped into similar categories, young and elderly volunteers that belong to the same immunological category, respond to vaccination similarly.

In this study, we provide data that supports the hypothesis that individuals with breadth of pre-existing immunity against historical strains is a benefit resulting from immunization with the currently licensed split IIV influenza vaccines. The ability to seroconvert to the vaccine is a primary determinant in a rise in antibodies to past influenza variants and most likely to co-circulating variants in any given season. As such, it can be said that current licensed vaccines offer breadth of immunity, at least to strains from the past, or strains belonging to similar antigenic clades. Whether this elicited immunity can afford protection against drifting variants of the future is currently being determined through deep assessments of individuals with breadth identified in this study and will be addressed in future analyses. The mechanism behind this back-boosting phenomenon is currently unknown, but most likely is not due to the production of novel antibodies with extensive cross-reactivity, but rather to recalling of memory B cells that results in the rise of anti-influenza HA antibodies against many epitopes and viral variants [46]. As such, influenza exposure history by infection, and possibly through vaccination, influences the breadth of immunity provoked through annual vaccination.
